# Selective Kinase Inhibition Shows That Bur1 (Cdk9) Phosphorylates the Rpb1 Linker *In Vivo*

**DOI:** 10.1128/MCB.00602-18

**Published:** 2019-07-16

**Authors:** Yujin Chun, Yoo Jin Joo, Hyunsuk Suh, Gaëlle Batot, Christopher P. Hill, Tim Formosa, Stephen Buratowski

**Affiliations:** a Department of Biological Chemistry and Molecular Pharmacology, Harvard Medical School, Boston, Massachusetts, USA; b Department of Biochemistry, University of Utah School of Medicine, Salt Lake City, Utah, USA

**Keywords:** Cdk9, P-TEFb, RNA polymerase II, Rpb1, Spt6

## Abstract

Cyclin-dependent kinases play multiple roles in RNA polymerase II transcription. Cdk7/Kin28, Cdk9/Bur1, and Cdk12/Ctk1 phosphorylate the polymerase and other factors to drive the dynamic exchange of initiation and elongation complex components over the transcription cycle. We engineered strains of the yeast Saccharomyces cerevisiae for rapid, specific inactivation of individual kinases by addition of a covalent inhibitor. While effective, the sensitized kinases can display some idiosyncrasies, and inhibition can be surprisingly transient. As expected, inhibition of Cdk7/Kin28 blocked phosphorylation of the Rpb1 C-terminal domain heptad repeats at serines 5 and 7, the known target sites. However, serine 2 phosphorylation was also abrogated, supporting an obligatory sequential phosphorylation mechanism. Consistent with our previous results using gene deletions, Cdk12/Ctk1 is the predominant kinase responsible for serine 2 phosphorylation. Phosphorylation of the Rpb1 linker enhances binding of the Spt6 tandem SH2 domain, and here we show that Bur1/Cdk9 is the kinase responsible for these modifications *in vivo*.

## Abstract (precis)

Cyclin-dependent kinases play multiple roles in RNA polymerase II transcription. Cdk7/Kin28, Cdk9/Bur1, and Cdk12/Ctk1 phosphorylate the polymerase and other factors to drive the dynamic exchange of initiation and elongation complex components over the transcription cycle. We engineered strains of the yeast Saccharomyces cerevisiae for rapid, specific inactivation of individual kinases by addition of a covalent inhibitor.

## INTRODUCTION

Phosphorylation provides a powerful mechanism for rapidly and reversibly affecting protein functions and interactions, and a very large percentage of eukaryotic proteins are phosphorylated *in vivo*. Accordingly, there is great interest in chemical kinase inhibitors, both for scientific studies and as potential drugs. The greatest challenge is to create compounds with sufficient specificity to overcome the structural similarities among kinase active sites. One powerful strategy is the “bump-hole” system, in which a “gatekeeper” residue in the target kinase ATP-binding pocket is mutated to create space for an ATP mimic carrying a bulky side group that blocks its binding to other kinases (1). Such mutated kinases are referred to as “altered specificity” (AS). This approach can be extended by engineering a reactive group on the inhibitor such that it covalently links to a specifically positioned cysteine found on the target kinase. Such covalent inhibitors are irreversible, which can produce more complete kinase inactivation. Using these approaches, an “irreversibly sensitized” (IS) kinase allele can be created with two point mutations (2–4).

A great deal of effort has gone into understanding the functions of the cyclin-dependent kinases (CDKs) involved in transcription by RNA polymerase II (reviewed in references 5 to 7). Cdk7/Kin28 assembles into the preinitiation complex (PIC) as part of the basal transcription factor TFIIH. It phosphorylates the C-terminal domain (CTD) of polymerase subunit Rpb1, primarily on serine 5 of the repeated consensus sequence YSPTSPS. This phosphorylation triggers several events near 5′ ends of genes: it dissociates the Mediator complex that chaperones RNA polymerase II (RNApII) into the PIC, while creating binding sites for the mRNA capping enzyme, the Nrd1/Nab3 early termination complex, and the Set1/COMPASS histone methyltransferase. As RNApII proceeds into elongation, two additional CDKs come into play. Cdk9/Bur1 phosphorylates the C-terminal repeat region (CTR) of the Spt5/DSIF elongation factor, which in turn promotes binding of the PAF1 complex (PAFc), another important elongation factor. In some eukaryotes, Cdk9/Bur1 also phosphorylates the negative elongation factor NELF to reverse its inhibitory effects. Cdk9/Bur1 may also phosphorylate serine 2 of the Rpb1 CTD at a low level during early elongation (8), although other experiments suggest that it also targets Ser5 (reviewed in reference 6). In any case, the vast majority of CTD Ser2 phosphorylation is due to Cdk12/Ctk1 during productive elongation. Ser2 phosphorylation promotes binding of the histone methyltransferase Set2 and multiple polyadenylation/termination factors to RNApII complexes as they proceed downstream (5–7).

AS alleles have frequently been used to inhibit these transcription-related kinases in budding and fission yeasts (8–17) as well as mammalian cells (18–20). Chemical inhibitors that target the native mammalian kinases with various levels of specificity are available (21–23). Importantly, the Ansari lab (4) demonstrated that a Kin28-IS inhibition was much more effective than Kin28-AS inhibition in yeast cells, possibly because powerful drug efflux channels limit the concentrations of inhibitors that can be maintained *in vivo*. Therefore, AS allele inhibition experiments have produced conflicting conclusions and failed to reveal effects seen using IS allele inhibition. Here we extend the IS approach to Bur1/Cdk9 and Ctk1/Cdk12.

We created double point mutations that confer sensitivity to the chemical CMK (so called due to its reactive chloromethyl ketone group), which has both a “bump” that can be accommodated by a “hole” mutation in the ATP-binding pocket and the CMK functional group that covalently cross-links to an engineered cysteine near the active site (2, 3). We found that the double mutants are expressed and functional, although they may not have full wild-type (WT) activity or stability. Inhibition *in vivo* was very rapid but surprisingly transient in liquid cultures, demonstrating the need for choosing an appropriate time point after CMK addition. As predicted, Kin28/Cdk7 inhibition reduced Ser5P and Ser7P, while Ctk1/Cdk12 inhibition blocked Ser2P. In contrast to most previous reports (see Discussion), we found that Ser2P was also strongly blocked upon Kin28 inhibition, indicating clear sequential dependence of the two marks. Bur1 inhibition also reduced CTD Ser2 phosphorylation, but less than Ctk1 inhibition, supporting our earlier findings that Bur1/Cdk9 is not the major Ser2P kinase (24). However, we discovered that Bur1/Cdk9 phosphorylates the Rpb1 linker region, a domain that lies between the RNApII body and the CTD. Phosphorylation of specific Rpb1 linker residues enhances binding of the Spt6 tandem SH2 (tSH2) domain (25, 26), indicating that Bur1/Cdk9 activity is important for functionally linking both elongation factors Spt5/DSIF and Spt6 to the elongating RNApII.

## RESULTS

### Creation of irreversibly sensitized kinase alleles.

Cohen et al. (2) created the covalent kinase inhibitor CMK as an inhibitor of ribosomal S6 kinase (RSK) and Polo-like kinase (PLK) family kinases. This molecule is an adenine-like pyrrolopyrimidine derivative that carries both a bulky “bump” constituent and a chloromethylketone group that covalently links to a reactive cysteine found in this family of kinases (2, 3). Rodriguez-Molina et al. (4) showed that CMK sensitivity could be conferred on a Kin28 mutant combining “hole” (L83G) and cysteine (V21C) mutations.

**FIG 1:**
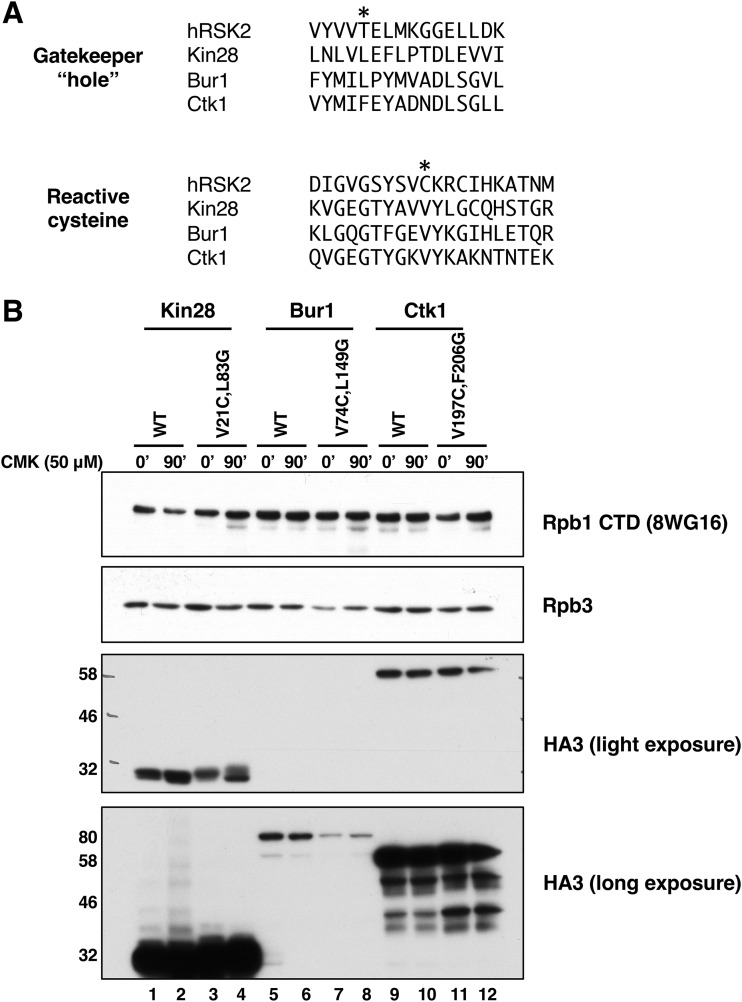
Construction of irreversibly sensitized (IS) kinase strains. (A) Sequence alignments of IS mutant positions (2–4) in human RSK2, Kin28, Bur1, and Ctk1 kinases. The residues mutated to create the “hole” or reactive cysteine are marked by asterisks. (B) Protein expression levels of wild-type and IS mutant kinases, before (0 min) or after 90 min of treatment with 50 μM CMK. Anti-HA blots show epitope-tagged kinases at the expected sizes of Kin28 (35 kDa), Bur1 (74 kDa), and Ctk1 (61 kDa). Rpb1 and Rpb3 are two RNA polymerase II subunits used as loading control bands. Strains used: YSB3216 (Kin28 WT), YSB3221 (Kin28 V21C, L83G), YSB3229 (Bur1 WT), YSB3232 (Bur1 V74C, L149G), YSB3235 (Ctk1 WT), and YSB3237 (Ctk1 V197C, F206G).

Using alignments of the Kin28/Cdk7, Bur1/Cdk9, and Ctk1/Cdk12 sequences, we designed corresponding “hole” and reactive-cysteine mutants for Bur1 and Ctk1 (Fig. 1A). The previously described Bur1 AS mutation L149G (8, 27) creates the “hole,” while changing valine 74 to cysteine (V74C) creates the covalent linkage site. Similarly, the combination of F260G and V197C mutations are predicted to create a Ctk1-IS protein. The mutated genes were introduced into yeast using plasmid shuffling, and protein expression levels were tested using a triple hemagglutinin (HA3) tag introduced onto the C terminus. Immunoblotting showed that Kin28-IS and Ctk1-IS proteins were expressed at levels similar to their wild-type counterparts (Fig. 1B). It should be noted that, unlike *KIN28* and *BUR1*, *CTK1* is a nonessential gene, so retention of the Ctk1-IS plasmid requires use of a selective growth medium. Bur1 levels are significantly lower than those of the other two kinases, necessitating a longer exposure for detection (Fig. 1B, bottom panel). Bur1-IS protein expression was lower than that of the wild type, indicating that the dual mutations affected its stability. Each single mutation also caused some reduction, apparently contributing additively in the double mutant (see Fig. S1A in the supplemental material). Bur1-IS levels could be boosted above normal wild-type levels by expressing the mutant on a high-copy-number plasmid (Fig. S1B), but these cells grew noticeably slower than the wild type and so were not used here. Treatment of cells with CMK did not affect levels of any of the kinases (Fig. 1B).

**FIG 2:**
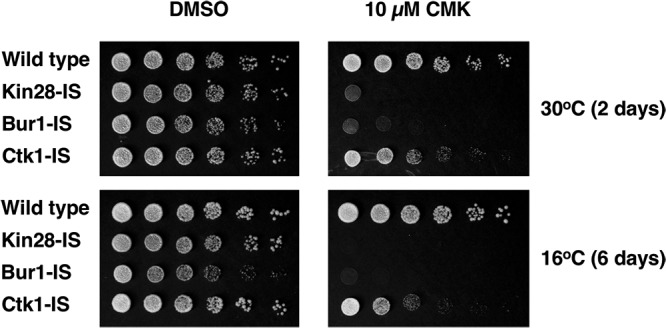
Growth inhibition of IS strains on CMK plates. Strains YF702 (wild type), YSB3356 (Kin28-IS), YSB3419 (Bur1-IS), and YSB3444 (Ctk1-IS) were spotted on YPD plates containing 10 μM CMK. Each row shows 3-fold dilutions. Plates were photographed after the indicated number of days at the indicated temperature.

The growth rates of IS strains at 30°C were similar to that of a wild-type control, indicating that the mutated kinases were functional. Bur1-IS grew slightly slower than the WT at 16°C, possibly reflecting the reduced protein levels. While wild-type cells were unaffected, both Kin28-IS and Bur1-IS cells showed strong inhibition of growth by CMK in plate spotting assays (Fig. 2). Ctk1-IS cells produced smaller colonies on CMK plates, particularly at 16°C, consistent with the phenotypes of *ctk1Δ* cells. These results indicate that CMK enters cells and inhibits the target kinases.

### Inhibition by CMK is effective but transient in liquid cultures.

**FIG 3:**
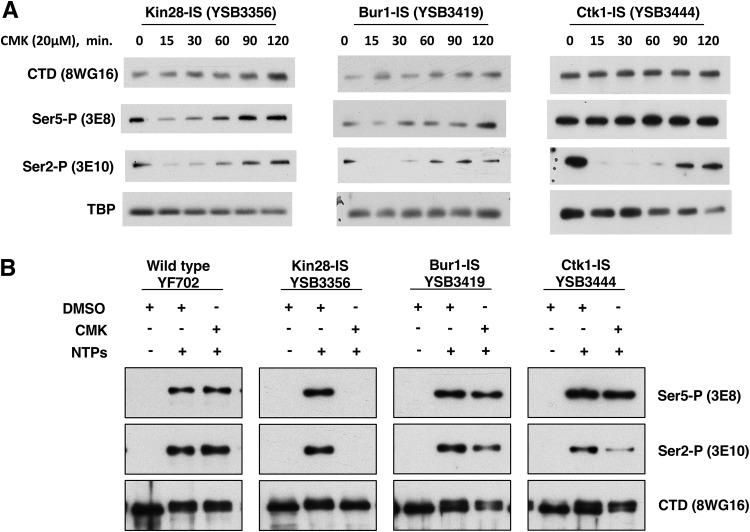
Inhibition of CTD phosphorylations in the IS kinase strains. (A) Time course of CTD phosphorylation levels during CMK inhibition *in vivo*. The indicated yeast strains were grown to mid-log phase (OD of 1.0) at 30°C. Immediately after taking a sample to serve as the zero time point, CMK was added to a 20 μM final concentration. At each indicated time point, samples were taken and processed for immunoblotting as described in Materials and Methods section. Blots were probed with the indicated antibodies. (B) Transcription complexes assembled *in vitro*. Yeast nuclear extracts were prepared from the indicated strains and incubated with a DNA template carrying five Gal4-binding sites upstream of the CYC1 core promoter and a G-less cassette. Immobilized templates were isolated and analyzed by immunoblotting as described previously (28).The first lane in each set shows RNApII preinitiation complexes formed in the absence of NTPs. The second and third lanes show elongation complexes formed upon treatment with ATP, UTP, and CTP for 4 min, with the third lane showing the effect of CMK inhibition. The first and second lanes also received DMSO to control for the CMK vehicle.

Given the clear growth inhibition on CMK plates, we were surprised to find that growth rates of the Bur1-IS and Ctk1-IS strains in liquid medium were not strongly affected by CMK. In our hands, CMK concentrations between 1 and 50 μM failed to completely arrest growth in rich or minimal medium liquid cultures. This observation contrasted with the case for Kin28-IS, which gave a strong growth arrest, as previously reported (4). The growth in CMK liquid medium was not due to revertants or suppressors, as subsequent plating on CMK solid medium still showed strong growth inhibition (not shown). To probe this discrepancy and to validate that CMK was effectively inhibiting the kinases, CTD phosphorylation was monitored using immunoblotting (Fig. 3A).

**FIG 4:**
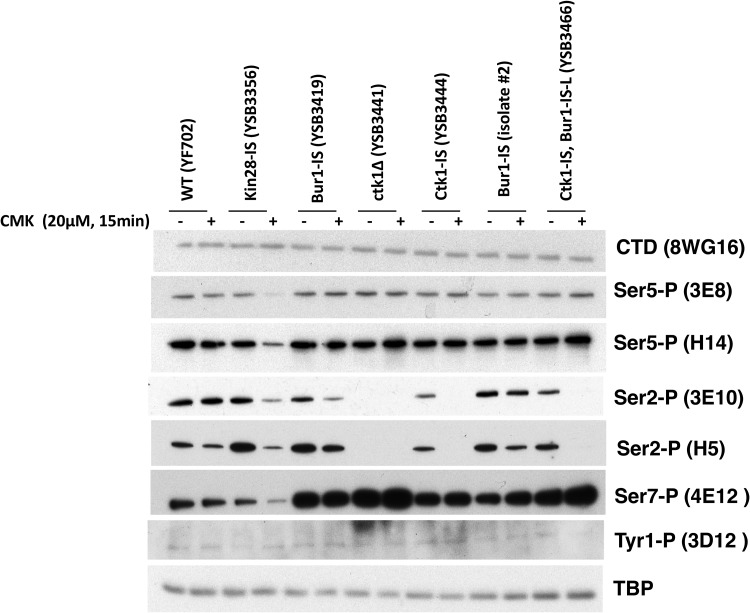
Analysis of IS kinase inhibition with different CTD antibodies. The indicated yeast strains were analyzed before or after 15 min of inhibition with 20 μM CMK. Blots were probed with the indicated antibodies. TATA-binding protein (TBP) is shown as a loading control. Note that all blots were developed with Pierce SuperSignal West Pico chemiluminescent substrate, except for 4E12 (Ser7-P) and 3D12 (Tyr1-P), which required the Femto maximum-sensitivity substrate. No signal above background was detected with 6G7 (Thr4-P) (not shown). Results from combined multiple experiments are quantitated in Fig. S2 in the supplemental material.

Inhibition of each kinase gave a different response. Notably, time courses revealed that CMK inhibition of all three kinases was very rapid but transient. For example, a 15-min CMK treatment of Ctk1-IS cells caused very strong loss of Ser2P but no effect on Ser5P, as expected for the major Ser2 kinase. However, Ser2P showed partial recovery by 90 min (Fig. 3A). As assayed by multiple CTD antibodies, Ctk1-IS inhibition for 15 min mimics the phosphorylation patterns seen in a *ctk1Δ* strain (Fig. 4). Inhibition of Bur1 for 15 min also reduced Ser2P, although not as completely as Ctk1 inhibition (Fig. 3A and 4). These results show that the time frame for maximal effect of these covalent inhibitors must be established and that interpretations should be limited to that window. Given that the Saccharomyces cerevisiae cell cycle in rich medium is about 90 min, this transient response explains why CMK did not always arrest the growth of liquid cultures. Accordingly, we found that repeated doses of CMK every 20 to 30 min produced a stronger growth arrest in liquid medium (not shown).

As expected, treatment of the Kin28-IS strain with CMK led to a significant drop in CTD Ser5P. This was seen with both 3E8 and H14 antibodies (Fig. 3A and 4). Ser7P, another known target site for Kin28/Cdk7, was similarly reduced (Fig. 4). The remaining low level of these modifications may reflect incomplete Kin28 inhibition, Ser5P phosphorylation by a different kinase, or persistence of some Ser5P phosphorylated before CMK addition. In contrast to the findings of Rodriguez-Molina et al. (4) and multiple Kin28-AS studies (11, 12, 14, 15, 17), we found that Kin28-IS inhibition also strongly inhibited Ser2P formation, as assayed by either 3E10 or H5 antibodies (Fig. 3A and 4).

Like that in Ctk1-IS and Bur1-IS, inhibition of CTD phosphorylations in the Kin28-IS strain was also transient, with recovery apparent by 60 min after CMK addition (Fig. 3A). This was unexpected given the strong growth inhibition. This postinhibition phosphorylation may reflect Kin28 that eventually escapes the CMK effect or perhaps is the result of other nontranscription kinases that act as the cells are dying (see Discussion).

We confirmed the effects of kinase inhibition using yeast nuclear extracts to assemble RNApII complexes on DNA templates containing five Gal4-binding sites upstream of the *CYC1* core promoter and a G-less cassette (28). The bead-immobilized templates were incubated with IS strain extracts treated with CMK or the solvent dimethyl sulfoxide (DMSO). Complexes were recovered magnetically, and bound proteins were isolated and analyzed by gel electrophoresis and immunoblotting (Fig. 3B). Preinitiation complexes formed in the absence of nucleoside triphosphates (NTPs) have no CTD phosphorylation (Fig. 3B, first lane in each kinase set). Elongation complexes stalled at the end of the G-less cassette were formed by treatment with ATP, UTP, and CTP for 4 min (second and third lanes). As we previously reported (28), and in agreement with the *in vivo* results (Fig. 3A), Kin28 inhibition *in vitro* blocked both Ser5 and Ser2 phosphorylation. In contrast, Ctk1 inhibition specifically reduced Ser2P, as did Bur1 inhibition to a lesser extent. As seen *in vivo*, neither Ctk1 nor Bur1 reduced Ser5P levels. In general, the effects of CMK *in vitro* were more definitive than those in the *in vivo* experiments.

### Bur1 phosphorylates residues in the Rpb1 linker region.

In addition to the Rpb1 heptamer repeats, phosphorylations have been detected on several serines and threonines in the Rpb1 linker region just N terminal to the CTD (25, 26, 29). These residues are also likely substrates for cyclin-dependent kinases, as they are followed by proline. Like the CTD, the linker region is apparently flexible, as it was not apparent in earlier RNA polymerase II crystal structures. However, Rpb1 linker phosphorylation promotes interaction with the Spt6 elongation factor, via contacts between the Spt6 tSH2 domain and phosphorylated Rpb1 residues T1471 and S1493 (25). Interestingly, the phosphate on T1471 combines with nonphosphorylated Y1473 to occupy the pocket that recognizes phosphotyrosine in other SH2 proteins (25). A recent cryo-electron microscopy (cryo-EM) reconstruction of the mammalian elongation complex was able to dock a linker-tSH2 crystal structure into density near the Rpb6-Rpb7 interface (26).

**FIG 5:**
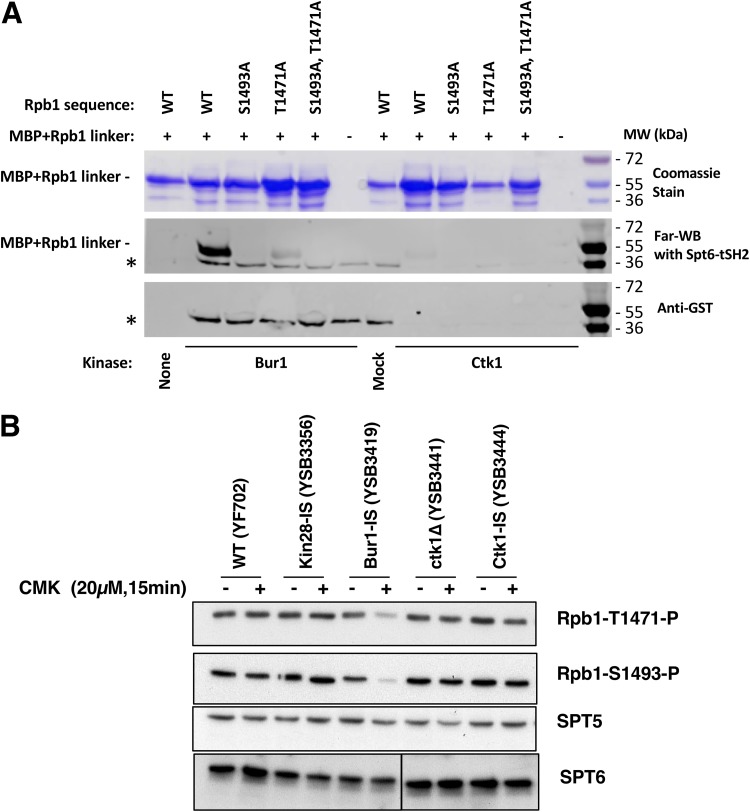
Bur1 phosphorylates Rpb1 linker region residues that mediate interaction with the Spt6 tSH2 domain. (A) Phosphorylation by Bur1 or Ctk1 enhances *in vitro* binding of Spt6-tSH2 to an Rpb1 linker peptide. MBP fusions to the Rpb1 linker (residues 1456 to 1511) or mutated derivatives thereof were purified and then incubated with partially purified His_12_-Bur1, His_12_-Ctk1, or the corresponding mock purification fractions from an untagged strain. Proteins were split and separated by SDS-PAGE. One aliquot was stained with Coomassie blue (top panel) to show the amounts and purity of the MBP-Rpb1 linker proteins. A second aliquot was transferred to nitrocellulose, refolded, and then probed with GST-Spt6 tSH2, which was subsequently detected using antibodies against GST (middle panel, Far-WB). Strong binding to the WT linker peptide was observed after treatment with Bur1, but this was diminished in mutants lacking the sites previously shown to be important for this interaction (25) (S1493 and T1471). Ctk1 also supported binding at a lower level. The asterisk denotes a species found in the Bur1, mock, and, to a lesser extent, Ctk1 preparations that directly bound the anti-GST antibody, as can be seen in a parallel blot with GST-Spt6 tSH2 protein omitted (bottom panel, anti-GST). (B) The indicated yeast strains were analyzed before or after 15 min of inhibition with 20 μM CMK. Blots were probed with the antibodies specific for Rpb1 linker sites (see Fig. S3B in the supplemental material) or Spt5 and Spt6 as loading controls. Note that the Spt6 bands are all from the same exposure of a single blot, but intervening lanes were removed.

The kinase responsible for these Rpb1 linker phosphorylations *in vivo* has not yet been identified. To address this question, a recombinant maltose-binding protein (MBP)–Rpb1 linker region fusion protein was phosphorylated with either Bur1 or Ctk1 and tested for *in vitro* binding to the Spt6 tSH2 domains using far-Western blotting (Fig. 5A; see Fig. S3A in the supplemental material). No binding was observed without phosphorylation of the linker (Fig. 5A, lanes None and Mock). Phosphorylation by Bur1 strongly stimulated linker binding to the tSH2, while Ctk1 phosphorylation produced a weak signal. Interaction was abolished when the sites of phosphorylation were mutated to alanine (Fig. 5A). Therefore, the Rpb1 linker sites can be phosphorylated by the Cdk kinases *in vitro*. In agreement, a recent paper showed that mammalian Cdk9 can phosphorylate the corresponding linker sites *in vitro*, although other kinases were not tested (26).

To identify which kinase phosphorylates the linker *in vivo*, extracts from the different IS kinase strains were immunoblotted with antibodies specific for the two phosphorylated residues that support Spt6-tSH2 binding (Fig. S3B). The results showed that Bur1/Cdk9 was the only required kinase. Both T1471 and S1493 phosphorylations were unaffected by Kin28-IS or Ctk1-IS inhibition but were strongly reduced by CMK treatment of the Bur1-IS strain (Fig. 5B). In contrast to the case for CTD Ser2P, the linker phosphorylations were not reduced by Kin28 or Ctk1 inhibition, suggesting that neither kinase is required for Bur1/Cdk9 recruitment or activity on the linker.

## DISCUSSION

To understand the role of an individual kinase, one must observe the effects of inactivating its function *in vivo*. There are multiple methods to accomplish this, each with its own advantages and disadvantages. Genes for nonessential kinases can be deleted, although care must then be taken that phenotypes do not become masked by suppressing mutations accumulated during long-term passaging. Temperature-sensitive (TS) alleles can be very informative, but products of these genes are often partially defective at permissive temperatures or remain partially functional at nonpermissive temperatures. Degron fusions can support normal function under permissive conditions, but loss of function may be slow or incomplete. Interpreting outcomes from these methods can also be complicated, as loss of the kinase protein can have secondary effects distinct from the loss of the kinase activity, such as destabilization of interacting factors. Chemical inhibitors can overcome these issues, as the protein remains present while kinase activity is ablated. However, this approach requires the availability of an inhibitor that completely inactivates the target kinase without affecting other kinases.

Here we applied the IS allele approach (2–4) to the three major transcription kinases, Kin28/Cdk7, Bur1/Cdk9, and Ctk1/Cdk12. This inhibitor approach significantly improves kinase specificity by combining the gatekeeper hole mutation with an additional cysteine substitution that allows irreversible inhibition through covalent linkage (Fig. 1A). We find that CMK inhibition of IS alleles is effective and informative. However, the two sensitizing mutations can affect kinase function or protein levels in the absence of the inhibitor (Fig. 1B). This is not unexpected, given that the “hole” mutation changes the shape of the ATP-binding pocket. We find that Ctk1-IS is expressed as well as wild-type Ctk1 protein, but lower levels of CTD Ser2P suggest that activity is reduced in the mutant. For Bur1, the IS protein is expressed at reduced levels, although these can be boosted by increasing gene dosage. Despite the decrease in Bur1-IS protein, phosphorylation of targets in the Rpb1 linker region and CTD appears to be normal in the absence of CMK inhibitor, indicating an adequate level of Bur1 activity. The IS kinase strategy therefore provides an important probe for kinase function *in vivo*, but effects on protein expression and activity prior to addition of the inhibitor must be monitored.

Although covalent modification of the kinases is presumably nonreversible, we found that the effects of CMK inhibition can be transient in yeast liquid cultures. Although growth of Kin28-IS or Bur1-IS on plates was completely arrested on solid medium containing CMK (Fig. 2), CMK did not fully arrest growth of Bur1-IS liquid cultures. Immunoblotting showed that CTD phosphorylation was effectively inhibited immediately after CMK addition, but at least partial recovery was generally seen by 60 to 90 min (Fig. 3). Given that CMK covalently links to the target kinase, it is unlikely that the inhibitor dissociates once bound. The very rapid recovery also rules out the outgrowth of rare inhibitor-resistant mutants. Most likely, CMK is depleted or inactivated as cells continue to synthesize more of the target kinase. Inhibitor binding is in kinetic competition with the drug export pumps of yeast cells and the lability of CMK. Similar behavior occurs in mammalian cells, as drug efflux pumps were recently shown to be at the root of resistance to the CDK7/12/13 inhibitor THZ1 (30). Continued production of the IS kinase, perhaps by translation of existing mRNA, may eventually exceed the effective intracellular concentration of inhibitor. This effect might explain the difference between liquid and solid medium assays, since the latter starts with a much lower density of cells and maintains a large reservoir of medium where no cells are present. Repeated dosing with CMK may improve growth arrest in liquid culture.

The previous characterization of Kin28-IS by Rodriguez-Molina et al. (4) nicely demonstrated the utility of kinase inhibition, but we note several differences with our results. First, they found that CMK inhibited Ser5P as assayed by the IgM monoclonal antibody H14, but not when using the IgG 3E8 monoclonal antibody. In our hands, CMK inhibits reactivity with both anti-Ser5P antibodies similarly (Fig. 4). We also observed stronger Ser7P inhibition. Some of these differences may represent strain background differences. However, their experiments generally assayed cells after 60 min of CMK treatment. In our hands, significant recovery of CTD phosphorylation was apparent by 60 min, at which point our results more closely resemble theirs. In our strains, 15 min was an optimal time for observing strong inhibition before any apparent recovery.

Another important observation is that *in vivo* inhibition of Kin28-IS produces strong inhibition of Ser2P, as well as Ser5P and Ser7P. The Ser2P drop was unexpected, as this phosphorylation was reported to be unaffected in multiple earlier studies using AS alleles of Kin28/Cdk7 in budding or fission yeasts (11, 12, 14, 15, 17), as well as using the same Kin28-IS allele used here by us (4). Mammalian Cdk7-AS cells showed a partial reduction in both Ser5P and Ser2P (19, 20), while inhibition of mammalian Cdk7 with the covalent kinase inhibitor THZ1 strongly blocks Ser5P and Ser2P (21, 31). However, THZ1 also inhibits the Ser2 kinase Cdk12, and a recent study using more-specific Cdk7 and Cdk12 inhibitors again failed to see a Ser2P drop upon Cdk7 inhibition (32). In the *in vivo* systems, complete kinase inhibition is likely difficult to achieve and maintain (Fig. 3A), but *in vitro* using transcription on immobilized templates, the dependence of Ser2P on Kin28/Cdk7 activity in yeast appears to be absolute (28) (Fig. 3B).

Given the well-characterized *in vitro* specificity of Kin28/Cdk7 for CTD Ser5 and Ser7, our CMK results suggest that efficient Ser2 phosphorylation depends on Kin28 first phosphorylating either the CTD or some other substrate. There are several possible mechanisms that could explain this obligatory sequence of phosphorylations. One is that phosphorylation by Kin28 simply makes the CTD accessible to a Ser2 kinase. For example, CTD Ser5 phosphorylation releases Mediator (33), which may otherwise block Ser2 kinases. A second model is that Ser5P promotes binding or activity of Ser2 kinases. *In vitro* experiments suggest that yeast Ctk1 (34), metazoan Cdk12 (35), and mammalian Cdk9 (6) more efficiently phosphorylate Ser2 on a CTD “primed” by prior phosphorylation at Ser5 or Ser7. Supporting a recruitment model, Qiu et al. (8) found that cross-linking of Bur1 to the *ARG1* gene was partially reduced when a *kin28-ts16* strain was shifted to a nonpermissive temperature. Although they did not actually show that Ser2P was reduced under these conditions, they speculated that Ser5P recruitment of Bur1 promotes Ser2P. Arguing against this model is our observation that Kin28-IS inhibition does not affect Bur1’s function in phosphorylating the Rpb1 linker region (Fig. 5B). Furthermore, we find that Bur1 is still recruited to *in vitro* elongation complexes after Kin28-IS inhibition (28). Yet another possible linkage mechanism is that Kin28 acts as a CDK-activating kinase (CAK) to phosphorylate Ctk1 or Bur1. Although the Cak1 kinase performs this function in budding yeast, Cdk7 phosphorylation of the Cdk9 T-loop has been reported in mammalian cells (19). Although the detailed mechanisms remain to be worked out, our demonstration that Kin28 activity is required for subsequent Ser2 phosphorylation helps explain the sequential nature of these two modifications.

Although *in vivo* inhibition of either Bur1 or Ctk1 caused a drop in CTD Ser2P, the loss in Ctk1-IS was far more complete (Fig. 4). Liu et al. (27) and Qiu et al. (8) observed little change in Ser2P upon inhibition of Bur1-AS unless it was combined with *ctk1Δ*, but because Ctk1-IS inhibition alone produced strong loss of Ser2P in our system, any additional effect of Bur1 inactivation was difficult to see (Fig. 4). These results echo our earlier conclusions that Ctk1/Cdk12 produces the vast majority of Ser2P (24, 36), although Bur1/Cdk9 may contribute a small amount of Ser2P early in elongation (8) (Fig. 3B). Mutations in genes for Bur1 or its associated cyclin Bur2 cause phenotypes and genetic interactions diagnostic of elongation defects, so the partial drop in Ser2P may be explained, at least in part, by inefficient elongation leading to reduced levels of elongation complexes.

In no case did we observe inhibition of the phospho-Tyr1 signal (Fig. 4). The immunoblot signal for this modification was very low, consistent with our mass spectrometry analysis showing very little phosphotyrosine on the yeast CTD *in vivo* (29). The identity of the putative Tyr1 kinase in yeast remains unknown.

Our finding that Bur1/Cdk9 phosphorylates the Rpb1 linker sites that contact Spt6 further links this kinase to promotion of transcription elongation. The other well-characterized Bur1 substrate is the C-terminal repeat region (CTR) of Spt5, where phosphorylation promotes binding of the PAF complex elongation factor (27, 37). Interestingly, cells lacking the Spt6 SH2 or Spt5 CTR are slow growing but viable, and recent crystal structures show that other domains within these two proteins make the key contacts with RNApII (26). Point mutations in the two Rpb1 linker phosphorylation sites only modestly affect Spt6 ChIP signals (25, 26). Therefore, Bur1/Cdk9 phosphorylations are not essential for Spt5 and Spt6 recruitment *per se* but more likely promote later steps in elongation complex maturation.

Although some groups have reported that RNApII cross-linking to transcribed genes is unaffected by deletion of *BUR2* (8, 38), it is hard to imagine how RNApII elongation could be normal given the very slow growth, elongation-related phenotypes, and defects in Paf1 complex recruitment in these cells. In contrast to these other reports, we observed a pronounced drop-off in the RNApII chromatin immunoprecipitation (ChIP) signal from 5′ to 3′ ends of genes in *bur2Δ* budding yeast (24). Similarly, recent experiments in Schizosaccharomyces pombe (17) showed that inhibition of a Cdk9-AS strain also caused a marked polar elongation defect. Finally, Cdk9 inhibition in mammalian cells blocks RNApII from escaping past the early pause site (39). Therefore, the preponderance of evidence indicates that Bur1/Cdk9 phosphorylates multiple substrates to promote efficient elongation.

In conclusion, we believe that covalent inhibition of the transcription-related CDKs can provide important information about their function. With the proper caveats and controls, the IS kinase alleles should prove to be useful tools for our future *in vivo* and *in vitro* experiments, and we look forward to sharing them with other labs for their work.

## MATERIALS AND METHODS

### Molecular genetics.

Plasmids encoding triple-hemagglutinin (HA3)-tagged kinases were previously described (24, 40). Mutations were made using inverse PCR-mediated mutagenesis (primer sequences are available upon request) and confirmed by DNA sequencing. The strains used for Fig. 1 and Fig. S1 in the supplemental material were constructed using standard plasmid shuffling (41). For other figures, strains with kinase IS alleles integrated at the natural chromosomal locus were constructed using the *delitto perfetto* method (42). Correct clones were identified using the CMK sensitivity phenotype, followed by sequencing of PCR fragments amplified from the chromosomal locus. Yeast strains are listed in Table 1. Cell growth was assayed by plating a series of 3-fold dilutions on YPD (1% yeast extract, 2% Bacto peptone, 2% glucose) plates containing 10 μM CMK. CMK stocks were made by dissolving powdered CMK (MedChem Express HY-52101) to 10 mM in dimethyl sulfoxide (DMSO).

**TABLE 1:** Yeast strains used in this study

Strain name	Genotype
9479	*MAT***a** *ura3-Δ0 leu2-Δ0 trp1-Δ2 his3 lys2-128*∂ *RPB1*(+100, KanMX)
9866-1-1	*MAT***a** *ura3-Δ0 leu2-Δ0 trp1-Δ2 his3 lys2-128*∂ *rpb1-T1471*(+100, KanMX)
9833-1-2	*MAT***a** *ura3-Δ0 leu2-Δ0 trp1-Δ2 his3 lys2-128∂ rpb1-Y1473A*(+100, KanMX)
9791-1-2	*MAT***a** *ura3-Δ0 leu2-Δ0 trp1-Δ2 his3 lys2-128∂ rpb1-S1493A*(+100, KanMX)
YF702	*MAT***a** *ura3-1 leu2-3,3112 trp1-1 his3-11,15 ade2-1 pep4*Δ::*HIS3 prb*Δ::*his3 prc1*Δ::*hisG*
YSB3356	*MAT***a** *ura3-1 leu2-3,3112 trp1-1 his3-11,15 ade2-1 pep4*Δ::*HIS3 prb*Δ::*his3 prc1*Δ::*hisG kin28*(L83G, V21C)
YSB3417	*MAT***a** *ura3-1 leu2-3,3112 trp1-1 his3-11,15 ade2-1 pep4*Δ::*HIS3 prb*Δ::*his3 prc1*Δ::*hisG bur1*Δ::KanMX [pRS315-BUR1-HA3]
YSB3418	*MAT***a** *ura3-1 leu2-3,3112 trp1-1 his3-11,15 ade2-1 pep4*Δ::*HIS3 prb*Δ::*his3 prc1*Δ::*hisG bur1*Δ::KanMX [pRS315-BUR1(L149G)-HA3]
YSB3419	*MAT***a** *ura3-1 leu2-3,3112 trp1-1 his3-11,15 ade2-1 pep4*Δ::*HIS3 prb*Δ::*his3 prc1*Δ::*hisG bur1*Δ::KanMX [pRS315-BUR1(V74C, L149G)-HA3]
YSB3441	*MAT***a** *ura3-1 leu2-3,3112 trp1-1 his3-11,15 ade2-1 pep4*Δ::*HIS3 prb*Δ::*his3 prc1*Δ::*hisG ctk1*Δ::CORE-KI URA3
YSB3444	*MAT***a** *ura3-1 leu2-3,3112 trp1-1 his3-11,15 ade2-1 pep4*Δ::*HIS3 prb*Δ::*his3 prc1*Δ::*hisG ctk1*(V197C, F260G)
YSB3466	*MAT***a** *ura3-1 leu2-3,3112 trp1-1 his3-11,15 ade2-1 pep4*Δ::*HIS3 prb*Δ::*his3 prc1*Δ::*hisG ctk1*(V197C, F260G) *bur1*Δ::KanMX [pRS315-BUR1(V74C, L149G)-HA3]
YSB3216	*MAT***a** *ura3-1 leu2-3,3112 trp1-1 his3-11,15 lys2Δ202 kin28*Δ::*LEU2* [pRS314-haKin28]
YSB3221	*MAT***a** *ura3-1 leu2-3,3112 trp1-1 his3-11,15 lys2Δ202 kin28*Δ::*LEU2* [pRS314-haKin28 (L83G, V21C)]
YSB3229	*MAT***a** *ura3-52 leu2Δ1 trp1Δ63 his3Δ200 lys2Δ202 bur1*Δ::*HIS3* [pRS315-BUR1-HA3]
YSB3230	*MAT***a** *ura3-52 leu2Δ1 trp1Δ63 his3Δ200 lys2Δ202 bur1*Δ::*HIS3* [pRS315-BUR1(V74C)-HA3]
YSB3231	*MAT***a** *ura3-52 leu2Δ1 trp1Δ63 his3Δ200 lys2Δ202 bur1*Δ::*HIS3* [pRS315-BUR1(L149G)-HA3]
YSB3232	*MAT***a** *ura3-52 leu2Δ1 trp1Δ63 his3Δ200 lys2Δ202 bur1*Δ::*HIS3* [pRS315-BUR1(V74C, L149G)-HA3]
YSB3325	*MAT***a** *ura3-52 leu2Δ1 trp1Δ63 his3Δ200 lys2Δ202 bur1*Δ::*HIS3* [pRS425-BUR1(V74C, L149G)-HA3]
YSB3235	*MAT***a** *ura3Δ0 leu2Δ0 his3-11,15 met15Δ0 ctk1*Δ::KanMX [pRS313-CTK1-HA3-SSN6]
YSB3237	*MAT***a** *ura3Δ0 leu2Δ0 his3-11,15 met15Δ0 ctk1*Δ::KanMX [pRS313-CTK1(V197C, F260G)-HA3-SSN6]
7382-3-4	*MAT***a** *trp1 leu2 ura3 can1 pep4 prb1 his7*
MAS022	*MAT***a** *trp1 leu2 ura3 can1 pep4 prb1 his7 CTK1*-PP-6×Gly-12×His::KanMX
MAS023	*MAT***a** *trp1 leu2 ura3 can1 pep4 prb1 his7 BUR1*-PP-6×Gly-12×His::KanMX

### Antibody creation.

Polyclonal antiserum that specifically recognizes Rpb1 pS1493 was previously described (25). Polyclonal antisera against Rpb1 pT1471 and pY1473 were produced by Covance. Briefly, rabbits UT765 and UT768 were injected with the Rpb1 linker-derived peptide CGQDGGVTP(pY)SNESGLVN conjugated to keyhole limpet hemocyanin (KLH), and rabbits UT763 and UT764 were injected with the Rpb1 linker-derived peptide CEDGQDGGV(pT)PYSNESGL conjugated to KLH. The exsanguination bleed was subjected to positive and negative affinity purification steps over columns consisting of the phosphorylated peptide or unphosphorylated peptide, respectively. The specificity for the phosphorylated peptide versus the unphosphorylated peptide was validated by Western blots using WT strains or mutants with alanine substitutions at the modification sites (see Fig. S3B in the supplemental material).

### Immunoblotting.

The indicated yeast strains were grown to logarithmic phase (optical density at 595 nm [OD_595_] of ∼1.0) in standard YPD medium (except for Fig. 1, for which synthetic complete medium with appropriate omissions of amino acids for plasmid marker selection was used). Cells were collected by centrifugation and lysed with glass beads using either the trichloroacetic acid (TCA) method (43) or lysis buffer (50 mM Tris-HCl [pH 8.0], 150 mM sodium chloride, and 0.1% Nonidet-P40, supplemented with 1 mM phenylmethylsulfonyl fluoride [PMSF], 1 μg/ml leupeptin, 1 μg/ml pepstatin A, and 1 μg/ml aprotinin) as described previously (24, 40). Protein concentrations in lysates were determined using the Bio-Rad DC or Pierce Coomassie protein assay reagents, and 30 to 50 μg of total proteins per lane was separated by SDS-PAGE on 8% or 10% polyacrylamide gels, depending on the sizes of the proteins to be imaged. Proteins were transferred to nitrocellulose membranes and probed with the indicated antibodies. Rpb1 linker phosphorylations were assayed with anti-pT1471 (1:10,000), anti-pY1473 (1:500), anti-pS1493 (1:50,000), and anti-Rpb1-CTD (8WG16; 1:5,000) as described previously (25). Monoclonal anti-phospho-CTD antibodies were provided by Dirk Eick (6) (3E8, 3E10, 4D12, 4E12, and 6G7; all used at 1:1,000), generated in-house (H5, H14, 8WG16, and TATA-binding protein [TBP]; all at 1:1,000, except 1:2,500 for TBP), or purchased commercially (Rpb3 1Y26 from Neoclone and anti-HA 12013819001 from Roche; 1:1,000). For Fig. S3B, the secondary antibody was goat anti-rabbit serum coupled to an infrared tag (IR 800CW) for detection using a LiCor Odyssey instrument. For other figures, the secondary antibody was goat anti-rabbit (Sigma A0545) or goat anti-mouse (Jackson ImmunoResearch 115-035-044) antibody coupled to horseradish peroxidase for detection on film using Pierce SuperSignal West chemiluminescent substrate (Pico), except where noted, when the Femto maximum-sensitivity substrate was used.

### Immobilized-template experiments.

Transcription initiation and elongation complexes were assembled from yeast nuclear extracts onto bead-immobilized DNA templates as previously described (28). The template contains five Gal4-binding sites, the CYC1 core promoter, and a G-less cassette. After preincubation to form PICs, ATP, UTP, and CTP were added to allow transcription to the end of the G-less cassette. Stalled elongation complexes were then assayed by immunoblotting. Where noted, CMK (250 nM) or an equal volume of the DMSO vehicle only was added.

### Peptide binding far Western assay.

His-MBP-Rpb1 (residues 1456 to 1511) protein was diluted to 0.5 mg/ml in kinase buffer (25 mM HEPES [pH 7.5], 50 mM potassium acetate, 10 mM MgCl_2_, 10% glycerol, 2 mM dithiothreitol [DTT], 0.5 mM ATP), and 80-μl aliquots were prepared. Kinase complexes were prepared as described below, and 1.5 μl of each kinase was added to the MPB-Rpb1 and incubated at 30°C for 1 h. For blotting, 15 μl of reaction mixture was transferred to a tube containing 5 μl 4× SDS-loading dye. Five microliters of each sample was loaded on a 12% gel and electrophoresed for 1 h at 160 V. Far-Western blotting with glutathione *S*-transferase (GST)–tSH2, followed by anti-GST antibody, were performed as described previously (25). A parallel blot of the same samples was blotted with anti-GST directly to show which bands were dependent upon GST-tSH2.

### Protein expression and purification.

Wild-type Ctk1 and Bur1 complexes, carrying a PreScission protease-cleavable His_12_ tag fused to the target proteins, were purified from Saccharomyces cerevisiae strains MAS022 and MAS023, respectively. Eight liters of culture of each strain and a parallel culture of the parental strain 7382-3-4 lacking tagged kinases (for the mock sample) were grown in YPD inoculated with 50 ml of a saturated overnight culture and incubated at 30°C until they reached an OD of about 3. Cells were harvested by centrifugation, washed once with cold water, and pelleted by centrifugation. Cells were frozen by passing through a syringe into liquid nitrogen and lysed under liquid nitrogen using a Spex SamplePrep 6870 Freezer/Mill (Spex SamplePrep, Metuchen, NJ). Pulverized yeast was thawed in 2 pellet equivalents of lysis buffer (50 mM Tris-Cl [pH 7.5], 1 M NaCl, 10% glycerol, 40 mM imidazole, 1.4 mg/ml pepstatin, 1 mg/ml leupeptin, 1 mg/ml aprotinin, 1.9 mM PMSF). Lysates were clarified by centrifugation at 37,000 relative centrifugal force (RCF) for 30 min. The supernatant was incubated with 0.5 ml of Ni-nitrilotriacetic acid (NTA)–agarose (Qiagen) for 30 min at 4°C with agitation, followed by 3 washes with 5 ml of lysis buffer and then 2 washes with 5 ml of wash buffer (25 mM Tris-Cl [pH 7.5], 150 mM NaCl, 10% glycerol, 40 mM imidazole), and finally eluted with 4 times 2 ml of elution buffer (25 mM Tris-Cl [pH 7.5], 150 mM NaCl, 10% glycerol, 300 mM imidazole). The eluted protein was incubated with 20 μg PreScission protease in 6,000- to 8,000-molecular-weight-cutoff Fisherbrand regenerated cellulose dialysis tubing in dialysis buffer (50 mM Tris-Cl [pH 7.5], 500 mM NaCl, 10% glycerol, 15 mM imidazole, 2 mM β-mercaptoethanol [BME]) overnight. The protease was removed with glutathione-agarose. The subsequent flowthrough fraction was collected, and the resin was washed 2 times with 1 ml of dialysis buffer. The flowthrough and wash were pooled, concentrated to 200 μl using a 30-kDa Vivaspin concentrator (Sartorius Stedim Biotech, Aubagne, France), and flash frozen in liquid nitrogen prior to storage at 80°C. The negative-control strain was processed in parallel. Coomassie blue-stained gels of each preparation are shown in Fig. S3A.

GST-Spt6 tSH2 (residues 1247 to 1451) was expressed and purified as described previously (25) (Fig. 5A). His-MBP-Rpb1 (residues 1456 to 1511) and mutated derivatives were expressed from pET17B vectors (Invitrogen) in BL21 CodonPlus (RIL) Escherichia coli cells (Stratagene). Cultures were grown in 2 liters of autoinduction medium (44) in baffled 1.8-liter flasks at 37°C with continuous shaking. After 8 h, the cultures were shifted to 19°C and shaken for an additional 16 to 24 h. Harvested cells were stored at −80°C. Cells were thawed and lysed in buffer containing lysozyme, DNase, and protease inhibitors, followed by sonication. Lysates were clarified by centrifugation at 15,000 rpm for 35 min. The supernatant was incubated with 6 ml of Ni-NTA–agarose (Qiagen) for 30 min at 4°C with agitation, followed by 3 washes with 25 ml of lysis buffer and then 2 washes with 15 ml of wash, and finally eluted 2 times with 20 ml of elution buffer.

## Supplementary Material

Supplemental file 1
