# Supplementary material for: Selective Kinase Inhibition Shows That Bur1 (Cdk9) Phosphorylates the Rpb1 Linker In Vivo
Source: Mol Cell Biol. 2019 Jul 16;39(15):e00602-18. doi: 10.1128/MCB.00602-18 (PMC6639251; doi:10.1128/MCB.00602-18)
Supplement: Supplemental file 1 [file MCB.00602-18-s0001.pdf]

Supplemental Figures for Chun et al.

Selective kinase inhibition shows that Bur1 (Cdk9) phosphorylates the Rpb1 linker in vivo

Sup Fig 1. Bur1 IS mutations affect protein levels.

A. Strains carrying the indicated Bur1 single or double mutations were assayed for protein levels by probing for the HA-epitope tag. Strains used are YSB3229 (WT), YSB3230 (V74C), YSB3231 (L149G), and YSB3232 (V74C, L149G). A non-specific band around 43 kDa acts as a loading control.

B. Levels of Bur1-IS protein can be boosted by high copy expression. Cell extracts were analyzed as in part A. Strains used are YSB3229 (WT), YSB3232 (V74C, L149G, CEN), and two isolates of YSB3325 (V74C, L149G, 2μ). In addition to two different exposures, a photograph of the Ponceau-stained blot is shown as a loading control.

Sup Fig 2. Bar graph compiling data from multiple inhibition experiments. Densitometry for the indicated phospho-epitopes was used to quantitate levels at the zero time point and after 15 minutes with 10 μM CMK. Bars indicate the ratio of these values, with error bars showing standard deviation.

Sup Fig 3. Kinases and antibodies used for analyzing Rpb1 linker region phosphorylations.

A. Coomassie stained gel of kinase preparations used in **Figure 5A**. Kinases were purified from yeast using his-tagged proteins as described in Methods. Double asterisk shows position of the kinase subunit, single asterisk shows positions of associated proteins (Bur2 in the case of Bur1; Ctk2 and Ctk3, which run near each other, for Ctk1). "Mock" shows background proteins from an untagged strain, used as a negative control for non-specific kinases associated with the beads.

B. Three independent cultures of WT (Rpb1) or mutant yeast strains with alanine substitutions at the three mapped Rpb1 linker phosphorylation sites (T1471, Y1473, and S1493) were tested with each phosphorylation-specific antibody. An antibody against the Rpb1 CTD and total protein staining are shown as loading controls. Signals from multiple experiments were quantified, normalized to the average signal from WT controls run on the same gel, then compiled in the panels on the right with the average and standard deviation shown. The rpb1-T1471A strain displayed reduced signal for phosphorylated T1471 as expected, but also produced less signal with the antibodies against phosphorylated Y1473, suggesting this threonine may be important for phosphorylation of Y1473. A similar but smaller effect was also seen with reduced phosphoT1471 signal in an rpb1-Y1473A strain. These changes could also reflect changes in the epitopes recognized by the antibodies.

Sup Fig 1

A

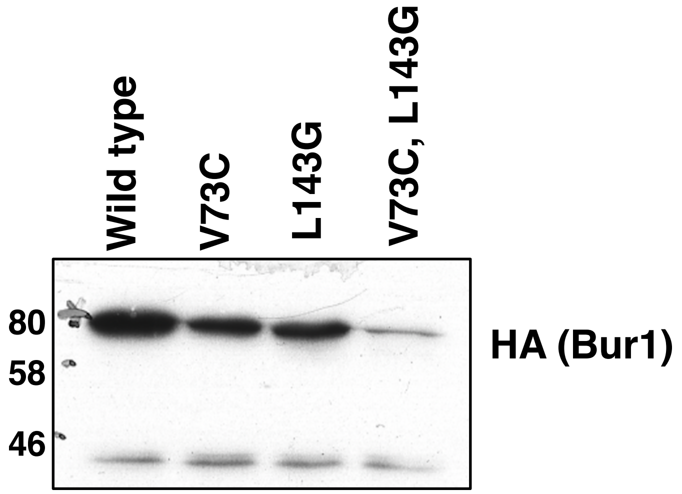
Wild type
V73C
L143G
V73C, L143G
80
58
46
HA (Bur1)

B

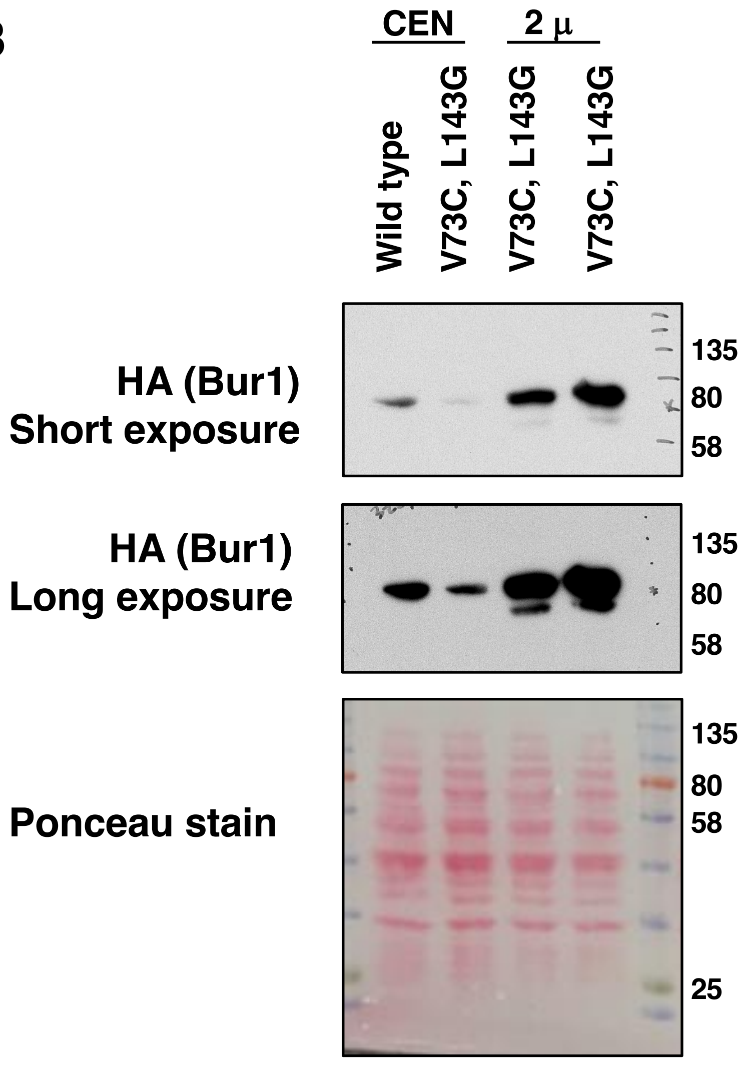
CEN
2 μ
Wild type
V73C, L143G
V73C, L143G
V73C, L143G
HA (Bur1)
Short exposure
135
80
58
HA (Bur1)
Long exposure
135
80
58
Ponceau stain
135
80
58
25

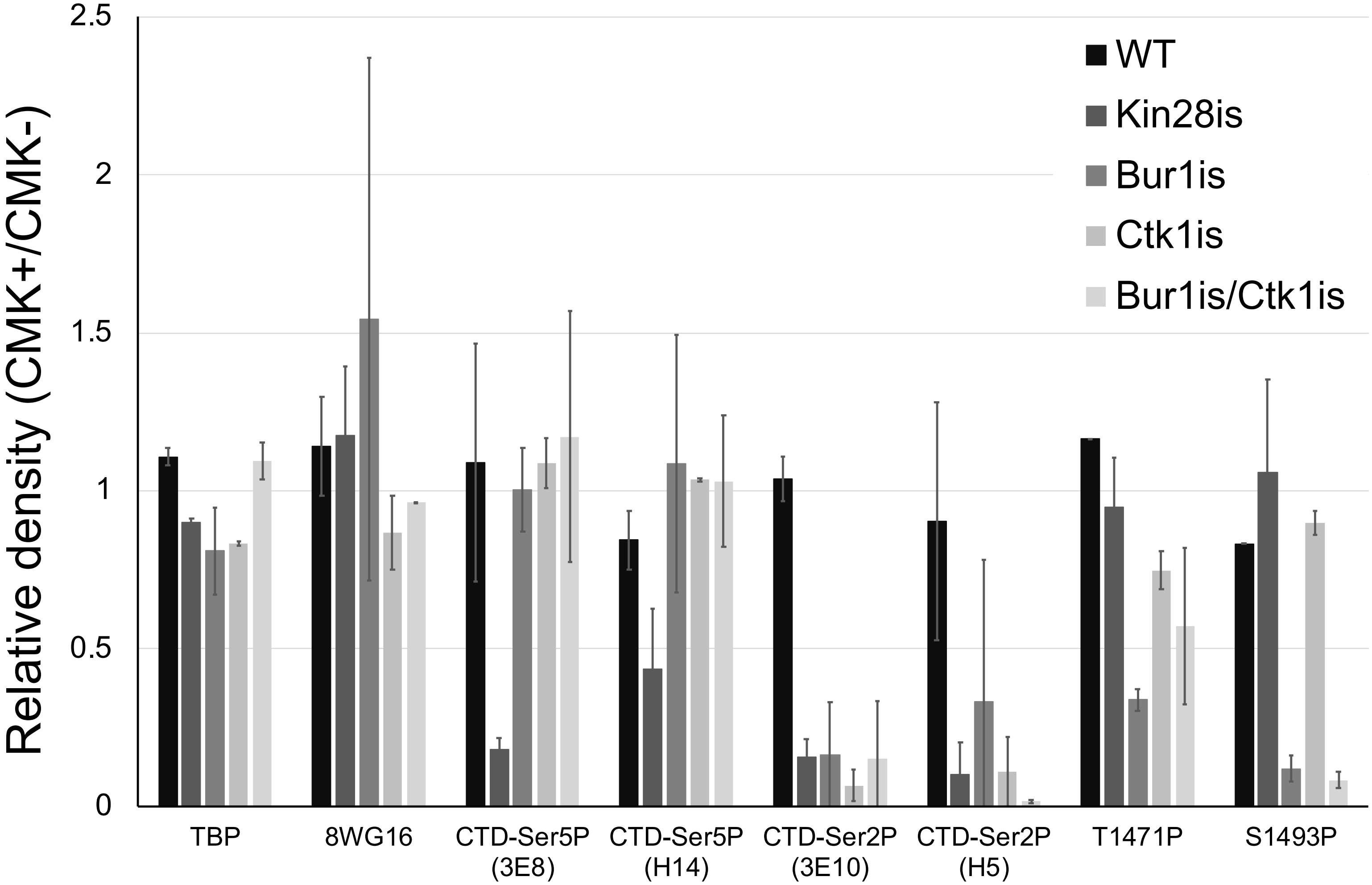
Relative density (CMK+/CMK-)
2.5
2
1.5
1
0.5
0
WT
Kin28is
Bur1is
Ctk1is
Bur1is/Ctk1is
TBP
8WG16
CTD-Ser5P (3E8)
CTD-Ser5P (H14)
CTD-Ser2P (3E10)
CTD-Ser2P (H5)
T1471P
S1493P

Sup Fig 3

A

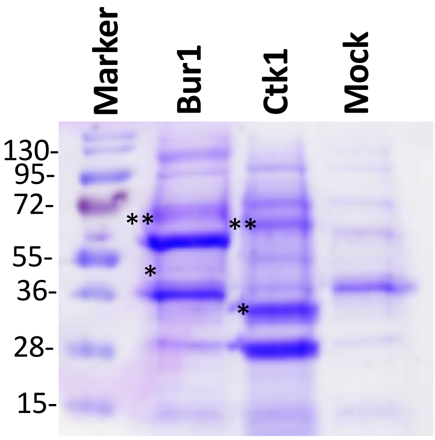

Marker
Bur1
Ctk1
Mock
130-
95-
72-
55-
36-
28-
15-
**
*
**
*

B

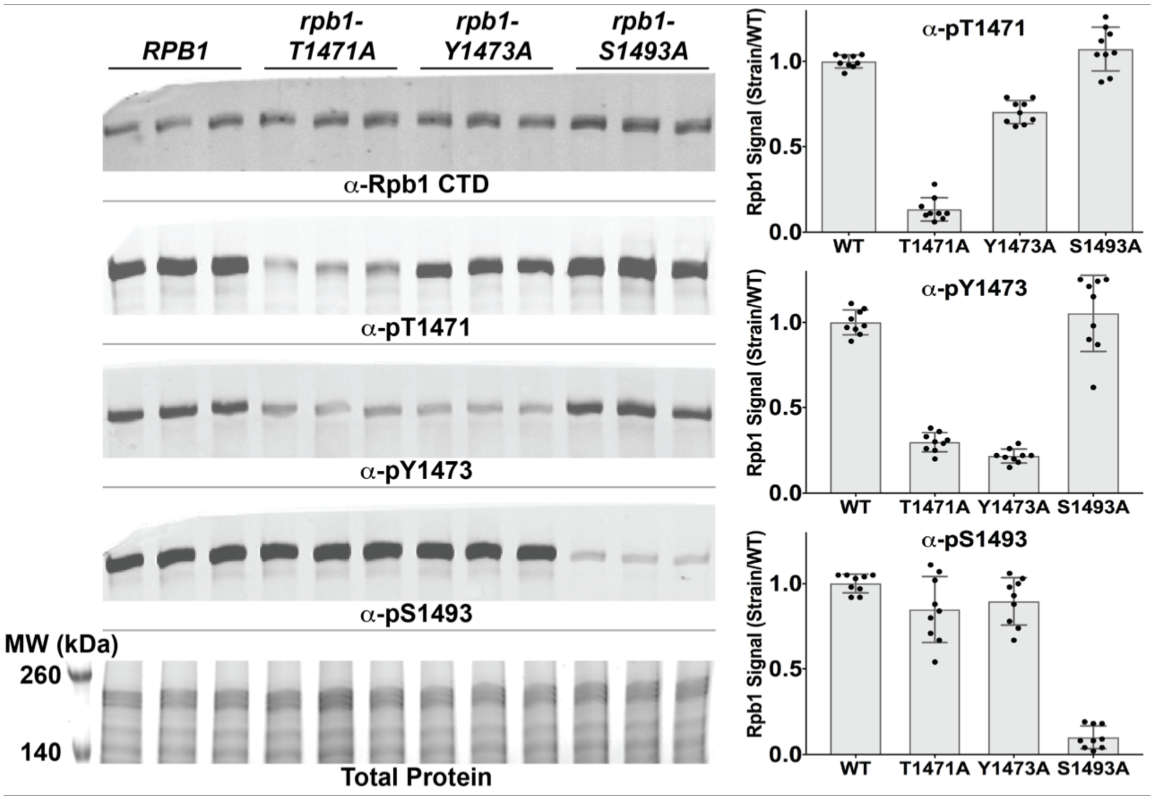

RPB1
rpb1-T1471A
rpb1-Y1473A
rpb1-S1493A
α-Rpb1 CTD
α-pT1471
α-pY1473
α-pS1493
MW (kDa)
260
140
Total Protein
α-pT1471
α-pY1473
α-pS1493
Rpb1 Signal (Strain/WT)
1.0
0.5
0.0
WT
T1471A
Y1473A
S1493A
